# Persistence of Daptomycin-Resistant and Vancomycin-Resistant Enterococci in Hospitalized Patients with Underlying Malignancies: A 7-Year Follow-Up Study

**DOI:** 10.3390/microorganisms12081676

**Published:** 2024-08-14

**Authors:** Lynn El Haddad, Georgios Angelidakis, Yuting Zhai, Layale Yaghi, Cesar A. Arias, Samuel A. Shelburne, Kwangcheol Casey Jeong, Roy F. Chemaly

**Affiliations:** 1 Department of Medicine, University of Florida, 2033 Mowry Rd, Gainesville, FL 32610, USA; 2 Department of Infectious Diseases, Infection Control and Employee Health, The University of Texas MD Anderson Cancer Center, Houston, TX 77030, USA; 3 Department of Animal Sciences, University of Florida, Gainesville, FL 32610, USA; 4 Emerging Pathogens Institute, University of Florida, Gainesville, FL 32610, USA; 5 Division of Infectious Diseases, Houston Methodist Hospital, Houston, TX 77030, USA; 6 Center for Infectious Diseases, Houston Methodist Research Institute, Houston, TX 77030, USA; 7 Department of Medicine, Weill Cornell Medical College, New York, NY 10065, USA

**Keywords:** vancomycin-resistant enterococci, immunocompromised, daptomycin, whole-genome sequencing, transmission

## Abstract

Vancomycin-resistant enterococci (VRE) commonly colonize the gut of individuals with hematologic malignancies or undergoing hematopoietic cell transplant (HCT) and may cause bacteremia. In 2012, we identified VRE isolates from patients and patients’ rooms and showed transmission networks of highly genetically related daptomycin-resistant (DR)-VRE strains. This is a follow-up study performing whole-genome sequencing (WGS) and phylogenetic analyses on 82 clinical VRE strains isolated from stools and blood cultures of patients with leukemia and HCT between 2015 and 2019. Here, we observed transmission of highly genetically related strains between rooms on the same or on different floors, including a DR-VRE strain identified in 2012. Eleven of twenty-eight patients with DR-VRE were never exposed to daptomycin, suggesting horizontal transmission. Fifteen of the twenty-eight patients with DR-VRE died within 30 days of positive blood cultures. Amongst those, one DR-VRE strain belonging to ST1471 had the virulence gene *bopD* responsible for biofilm formation. Additionally, to our knowledge, this is the first report of a DR-VRE strain belonging to ST323 in the United States. In summary, our study demonstrated the emergence and persistence of VRE strains, especially DR-VRE, in our hospital. Adding WGS to routine infection control measures may timely identify potential horizontal VRE transmission including multi-drug-resistant isolates.

## 1. Introduction

Vancomycin-resistant enterococci (VRE) are a major cause of healthcare-associated infections, with an estimated 54,500 cases among hospitalized patients and 5400 deaths per year associated with VRE in the United States alone [1]. In particular, patients with underlying hematologic malignancies such as leukemia and patients undergoing hematopoietic cell transplant (HCT) and solid organ transplant are at higher risk for VRE acquisition and infections due to their weakened immune system and multiple courses of antibiotics [2]. Contributing factors to the acquisition and spread of VRE include the prolonged use of broad-spectrum antibiotics, the gastrointestinal tract domination of VRE, prolonged hospitalization, and contact with a VRE-contaminated environment [3].

VRE colonization may lead to VRE bacteremia, which has significant morbidity and mortality [2]. A recent study by Contreras et al. followed patients with enterocoocal bloodstream infections in 11 hospitals across the US. The authors showed that 18% of those patients died and that the lack of VRE clearance was a significant predictor of in-hospital mortality [4]. VRE colonization rates vary widely among intensive care unit (ICU) patients, ranging from 2% in Finland to 34% in Ireland and 33% in the United States [5]. Hospital facilities may serve as a reservoir for VRE and are associated with a significant risk for VRE acquisition for hospitalized patients [3].

Daptomycin and linezolid are the current recommended options for the treatment of VRE infections [6]. According to surveillance data from 2009 through 2013, daptomycin demonstrated high sustained activity against VRE in Europe and the United States [7]. In fact, recent studies demonstrated improved outcomes in patients with VRE bloodstream infections when treated with daptomycin compared to linezolid [8]. However, the development of VRE isolates resistant to these antibiotics poses a serious clinical challenge, considering the findings of recent studies demonstrating high all-cause mortality (44.4%) associated with daptomycin-resistant (DR)-VRE infections and an overall 30-day mortality rate of 33% related to linezolid-resistant VRE infections [9].

In a previous study, we reported the presence and the transmission of highly related daptomycin-susceptible and DR-VRE isolates between HCT recipients and leukemia patients at a comprehensive cancer center using whole-genome sequencing (WGS) and single nucleotide polymorphism (SNP) analyses [3]. In this follow-up study, we compared the previously identified VRE sequence types (STs) from 2012 to newly isolated STs identified from 2016 to 2019 in the same patient population and hospital setting. Using WGS, we highlight the persistence of daptomycin- and linezolid-resistant VRE, the networks of transmission during that period, and the impact of VRE on patients’ outcomes.

## 2. Materials and Methods

### 2.1. Selection of VRE Isolates

A total of 82 consecutive clinical VRE strains, including 62 and 20 strains isolated from blood cultures and rectal swabs, respectively, were identified from patients with leukemia and HCT recipients housed in the intensive care unit (ICU), leukemia floors, or HCT floors at our comprehensive cancer center.

### 2.2. Data Collection

Data were retrieved from the infection control and pharmacy databases. Collected variables included room number, location (inpatient floor), date of VRE-positive culture (inpatient rectal swab or blood collection), VRE treatment courses (daptomycin, linezolid, and any other VRE-targeting antibiotics), prior exposure to daptomycin and linezolid up to 1 year prior to the isolation of VRE, source of isolation (blood culture or rectal swabs, indicating bacteremia or gastrointestinal colonization, respectively), prior rectal colonization for patients with VRE-positive blood cultures, 30-day outcomes, and date and cause of death. Bacteremia was defined as the isolation of bacteria from at least one blood culture in patients with signs and symptoms of an infection. Of interest, only one pair in this cohort consisted of a rectal swab strain and a blood strain isolated from the same patient.

### 2.3. Whole-Genome Sequencing and Assembly

DNA was extracted from the 82 VRE isolates using QIAamp DNA Stool Kit (Qiagen, Maryland, MD, USA). WGS was performed using Illumina MiSeq with 250-bp paired-end reads (Illumina Inc., San Diego, CA, USA). Sequencing adapters and low-quality bases were removed and genomes were assembled using SPAdes [10] and annotated using Prokka [11]. Core genomes were identified using Roary [12]. Phylogenetic trees were constructed using filtered core genome SNPs and the maximum likelihood tree search algorithm RAxML, and iTOL version 6 software was used to generate and visualize the tree.

### 2.4. MLST, Resistome, and Virulence Factors

VRE STs were determined using the MLST database (multi-locus sequence typing, https://pubmlst.org/organisms/enterococcus-faecium, accessed on 19 April 2024). Genes conferring resistance to the major antibiotics against enterococci were identified using the Resistance Gene Identifier (RGI) of The Comprehensive Antibiotic Resistance Database (CARD). Also examined was the LiaFSR regulon, a 3-component system that regulates cell-envelope stress response in *E*. *faecium* [13] and consists of a histidine kinase (LiaS), a membrane-bound protein (LiaF), and a response regulator (LiaR). For daptomycin resistance, amino-acid substitutions in predominant LiaFSR proteins were identified using BLAST+ v2.6.0 (including threonine to alanine in amino-acid position 120 of the LiaS histidine-kinase sequence and tryptophan to cysteine in position 73 of the LiaR response regulator) [13]. Linezolid resistance was identified based on mutations in the 23S rDNA genes and the presence of *optrA*, *poxtA*, and *cfr* (computational). Antibiograms of all strains were also reviewed from the microbiology reports when available. Microbiological testing available at the institution for isolating, identifying, and determining drug resistance includes disc diffusion assays and cultures on chromogenic media. When necessary, results may also be validated using MALDI-TOF. Virulence genes and their associated functions were identified using the Bacterial and Viral Bioinformatics Resource Center [14] by aligning the WGS of VRE strains against the virulence factor database (VFDB) [15] using BLASTN.

### 2.5. Transmission Networks

The identification of highly related VRE isolates and VRE transmission networks between patients on the different floors were stratified according to the number of SNPs found within the core genome of VRE isolates. Briefly, the core genome alignment file was processed with Snippy to identify the number of SNPs between all isolates [11]. We defined highly related strains as those differing by 5 SNPs or less. The 5-SNP threshold was selected because 26% of genomes were within 0–5 SNPs of 1–3 other isolates [16,17].

### 2.6. Data Availability

The data that support the findings of this study are publicly available on the National Center for Biotechnology Information platform under Bioproject PRJNA1009092.

## 3. Results

### 3.1. Overview of the VRE Strains

Eighty-two strains of VRE were collected from rectal swabs (n = 20) and blood samples (n = 62) between September 2016 and October 2019. Table 1 displays the 82 VRE strains according to their STs, culture types, and floors. All 82 VRE strains belonged to the species *Enterococcus faecium* and were recovered from leukemia and HCT recipients housed on different hospital floors: leukemia floors (n = 44 VRE isolates), HCT floors (n = 23), and ICUs (n = 15) (Table 1).

Of the 62 patients with VRE blood isolates, 42 (68%) had prior colonization with VRE before experiencing a bacteremia event. Of interest, the VRE pair isolated from a rectal swab strain and blood culture from the same patient were highly genetically related. The 82 strains belonged to the same clonal complex, CC17, and included 17 different STs. Most of the isolates had known STs (n = 79). Among those with known STs, 52 (66%) were found on all floors: ST17 (n = 20), ST18 (n = 13), ST664 (n = 8), ST736 (n = 7), and ST584 (n = 4). All 82 strains carried genes conferring resistance to common antibiotics. Most VRE strains were resistant to macrolides, lincosamides, streptogramin B (99%), teicoplanin (95%), kanamycin and neomycin (78%), and minocycline and tetracycline (77%) (Table 2).

### 3.2. Antibiotic Resistance and Virulence

**Figure 1:**
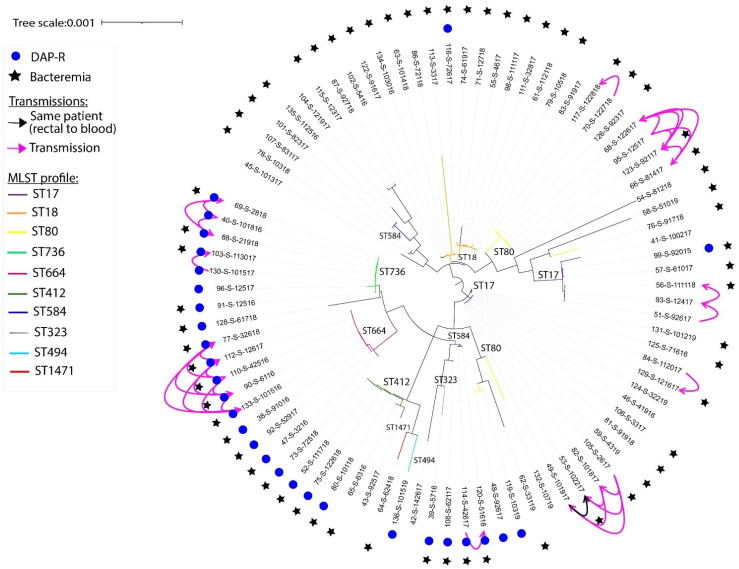
**Phylogenetic tree showing the genetic relatedness according to SNPs** found in the core genome of the 82 VRE isolates from 2016 to 2019. Transmissions between patients are shown. Phylogenetic trees were constructed using filtered core genome SNPs and the maximum likelihood tree search algorithm RAxML, and iTOL software was used to generate and visualize the tree. *Abbreviations:* ST, sequence type; DAP-R, daptomycin-resistant.

A total of 28 strains (34%) were resistant to daptomycin, identified both computationally and phenotypically (as described above). Computationally, these strains harbored both chromosomally encoded LiaR^W73C^ and LiaS^T120A^ substitutions, which confer daptomycin resistance (Table 2) [20]. Other mutations identified in three DR-VRE strains were located on the phospholipid biosynthesis enzyme cardiolipin synthase (*cls*) gene, including D27N and H215R [20,21]. The DR-VRE strains were isolated from 8 ICU patients, 9 HCT recipients, and 11 leukemia patients. Most of the DR-VRE strains belonged to either ST736 (8/28) or ST664 (7/28). These two STs were exclusively associated with daptomycin resistance in both our 2012 and 2015–2019 cohorts (Figure 1 and Figure S1). One pair consisted of a rectal swab strain and a blood strain isolated from the same patient and sharing high genetic relatedness (Figure 1). Additionally, all DR-VRE strains isolated from the ICU belonged to ST736 and were isolated from blood cultures. Of note, 13 of the 28 patients (46%) with DR-VRE were exposed to daptomycin for 1 to 22 days within 1 year of isolation of VRE; the remaining patients had no prior exposure to daptomycin within 1-year prior of VRE isolation, indicating a probable horizontal transmission between patients and between different floors. Among the 28 patients with DR-VRE strains, 22 patients died—15 within 30 days and 6 at a median of 3 months (interquartile range, 1–3 months) after positive blood cultures. One patient died 11 months after a DR-VRE strain was isolated from a rectal swab.

Linezolid resistance was identified in 11 VRE isolates in 7 leukemia patients and 4 HCT recipients; 7 out of 11 patients had a prior exposure to linezolid. The linezolid-resistant VRE strains belonged to ST80 (8/11), ST18 (1/11), ST17 (1/11), or ST1390 (1/11). One VRE strain (ST17) isolated from a leukemia patient in the ICU was both linezolid and daptomycin-resistant. The 82 VRE strains are clustered according to SNPs in their core genome. Their respective STs, antibiotic resistance, and source of isolation are shown in the phylogenetic trees in Figure 1, Figures S1 and S2; Figure S1 displays all isolates from 2012 to 2019.

Putative virulence genes were identified in both DR-VRE and non-DR-VRE isolates (including isolates that were linezolid resistant). Most of the DR-VRE and non-DR-VRE strains had genes responsible for bacterial adherence: *sgrA* (DR: 100% and non-DR: 94%), *esp* (DR: 97% and non-DR: 94%), *acm* (DR: 100% and non-DR: 97%), *ecbA* (DR: 48% and non-DR: 55%), *scm* (DR: 45% and non-DR: 71%), and *fss3* (DR: 97% and non-DR: 89%). Interestingly, the gene *bopD*, associated with biofilm formation, was detected only in a DR-VRE strain belonging to ST1471, isolated from a patient with persistent VRE bacteremia and septic thrombophlebitis.

### 3.3. Transmission

**Figure 2:**
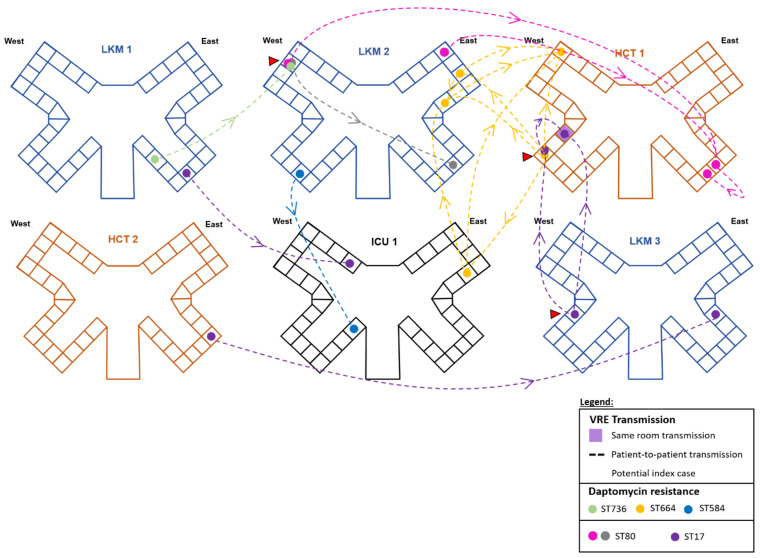
**Transmission networks of VRE isolates** differing by 5 or less SNPs in their core genomes during 2016 to 2019 in the hospital setting. Potential index cases of VRE are marked with a red triangle. The transmission time period ranged from 1 to 2 years with a median of 206 days. LKM, leukemia; HCT, hematopoietic cell transplant; ICU, intensive care unit. Note: There are 2 distinct clusters of ST80 VRE strains originating from the same room (different patients, different dates) and spread to patients on different floors, indicating possible recombination events and/or horizontal transmission.

A total of 22 of the VRE strains isolated from different hospital floors (leukemia, 9/22; HCT, 7/22; and ICU, 6/22) were highly genetically related, differing by five SNPs or less (Figure 2, Figures S3 and S4). These VRE strains belonged to ST17 (n = 6), ST80 (n = 6), ST664 (n = 5), ST736 (n = 3), or ST584 (n = 2). Around 36% of these potential horizontal transmissions were due to DR-VRE strains, with clonal isolation of ST736, ST664, and ST584 strains occurring between units and patients at a median of 206 days (range: 1 day to 2 years) after colonization or infection was found in a potential index patient (Figure 2).

When comparing VRE STs and transmission events identified in 2012 to those of the newly isolated VRE strains from 2016 to 2019, we observed the emergence of STs that were not found in the 2012 cohort, including ST80, ST117, ST323, ST431, ST789, ST282, ST233, ST1390, and ST1471. Of note, ST80 was identified in 11 of the 82 strains in the new cohort and was not detected in the 2012 cohort. One observation was the switch in ST494 from a daptomycin-susceptible VRE strain isolated in 2012 to a DR-VRE strain isolated in this new cohort on the HCT floor [3]. Resistance increased against all screened antibiotic classes, with considerable increases in minocycline and tetracycline as well as aminoglycosides resistance compared to the antibiotic susceptibility patterns recorded in 2012. Interestingly, when looking at all DR-VRE and linezolid-resistant VRE genomes, genes encoding resistance to aminoglycosides, streptogramin were located on plasmids carrying vancomycin-resistant genes whereas genes encoding resistance to tetracycline *tet(M)* genes were located on the chromosome.

In comparing the two cohorts, we observed a possible 5-year transmission in three of the DR-VRE strains (2012 to 2017 transmission). Indeed, one DR-VRE strain isolated in 2012 had less than five SNP differences compared to two of the 2017 DR-VRE strains, making them highly genetically related. Although this transmission could be initiated from different nosocomial or community-acquired routes, all three DR-VRE strains were isolated from the leukemia unit and belonged to ST736 (Figure S1, red transmission arrows). More importantly, all other VRE isolates were not related and indicate the ease of emergence and spread of VRE strains in the hospital.

## 4. Discussion

In this study, we used WGS and SNP analyses to assess horizontal transmission and identify antibiotic resistance and putative contributors to virulence among 82 VRE strains collected during 2016 to 2019 and compared these findings to those of our 2012 cohort [3]. Eight major VRE STs (i.e., ST17, ST18, ST736, ST664, ST412, ST584, ST203, and ST494) had persisted in hospitalized patients since 2012 and corresponded to 72% of the identified STs in the new cohort. These STs have spread worldwide throughout the years [22,23,24]. The remaining STs were all newly isolated in our facility and included ST80, ST117, ST323, ST431, ST789, ST282, ST233, ST1390, and ST1471. These latter STs, except for ST323, have been isolated in the US since 1996, as well as in different countries including France [22], Algeria [25], Portugal [24], Italy [26], China [27], Germany [9], and Australia [28]. DR-VRE STs of interest were ST323, identified for the first time in this study, and ST1471, harboring a gene conferring biofilm formation properties.

Studies have shown a major increase in DR-VRE strains in the US and worldwide [3,4,29]. In our study, only 46% of our patients with DR-VRE had daptomycin exposure within 1 year of VRE isolation whereas in a prior publication from our institution, 88% of leukemia patients with DR-VRE had daptomycin exposure for ≥13 days within 90 days preceding a bacteremia event, underscoring the impact of daptomycin exposure and occurrence of resistance in these high-risk patients [30]. In both the 2012 cohort and in our recent cohort, most of the DR-VRE isolates were either ST736 or ST664. Since its identification in 2014, ST736 and its association with daptomycin resistance has had major implications for infection control and patient management across hospitals and cancer centers [31]. Of note, VRE strains clustered under ST736 were identified predominantly in the ICUs in our recent cohort. Additionally, the identification of 5-year transmission of ST736 in our study underscores the persistence and expansion of this ST [3,20]. Indeed, studies have shown that VRE ST736 might have a unique genetic background predisposing these VRE strains to become daptomycin resistant and allowing dissemination in the hospital setting. This ST was identified in a New York hospital in 2014 before expanding throughout the United States and then worldwide to Canada, the Caribbean, Germany, and South America, raising public health concerns about its rapid dissemination [20,31].

Linezolid resistance was detected in 10 non-DR-VRE strains and in 1 DR-VRE. Interestingly, more than half of the linezolid-resistant VRE strains belonged to ST80, supporting the findings of other studies, and harbored the *optrA* gene or had mutations on the *eatAv* gene [32]. In fact, seven linezolid-resistant VRE strains had mutations on the *eatAv* gene, a member of the ATP-binding cassette F that has a role in protecting bacterial ribosomes from ribosome-targeting antibiotics such as linezolid [33]. One strain was harboring *optrA* and was not linked to any worsening clinical outcome. Strains carrying *optrA* emerged worldwide in 2015, but their prevalence remains low in clinical isolates in the United States, consistent with our findings [31,34]. The remaining three VRE strains had the point mutation G2604T within the 23s rDNA subunit previously shown to confer resistance to linezolid [35].

Three of the STs newly identified in our facility (ST80, ST323, and ST1471) were DR-VRE isolates. ST80, accounting for 13% of our VRE strains, was identified in 1927 in the United States but was not detected in the 2012 VRE cohort [3,36]. Although we have not typed all VRE strains isolated in our hospital, the finding of ST80 might indicate its possible emergence in our hospital setting. Studies have conflicting data concerning ST80 and its association with daptomycin resistance [4,37], suggesting that horizontal transmission may be occuring in addition to antimicrobial pressure. Interestingly, we report ST323 in the US for the first time, as it was previously identified only in China, France, Czech Republic, and Russia [38], highlighting the ease of global spread of VRE and other multi-drug-resistant organisms spread [39]. Additionally, ST1471 was first isolated in 2017 from a nonsterile preparation of injectable contrast used in interventional radiology, leading to an outbreak of VRE infections [17]. This daptomycin-resistant ST was isolated in our cohort in 2017 and was also identified in two other hospitals in Texas and Michigan between 2017 and 2018 [4]. On the other hand, in 2012, the VRE strain with ST494 was daptomycin susceptible compared to the DR-VRE strain with ST494 from our recent cohort, highlighting the emergence of DR-VRE and the potential of horizonal transmission of resistance genes in hospital settings.

A longitudinal study showed that VRE undergoes genomic rearrangemenets during intestinal colonization and bloodstream infections in immunocompromised patients, increasing VRE’s adherence to surfaces, biofilm production, and resistance to lysozyme and antibiotics [40]. Moreover, a study revealed that VRE strains isolated from hospital wastewater might have disseminated into the aquatic environment in South Africa, emphasizing the public health threat of VRE propagation to the community [41]. Those major concerns highlight the need for diagnostic improvement and detection of antibiotic resistance genes and mutations, to predict VRE susceptibility to drugs and manage the care of patients at risk of infections. WGS is a powerful tool to detect and uncover VRE resistance mechanisms and transmission patterns. WGS data can be used to predict antibiotic resistance by identifying the presence of these genes or SNPs that trigger antibiotic resistance, or by predicting the presence of specific genetic loci using statistical analyses and machine learning. These methods have been successfully used for *E*. *faecium* to predict antimicrobial resistance [42,43]. Another major use of WGS is the detection of potential outbreaks [17]. A recent study described an outbreak of VRE infections in a US hospital using WGS surveillance to discover a previously hidden cluster of VRE and to identify the potential source and route of transmission following interventional radiology procedures. WGS surveillance in this study linked non-sterile technique to the emergence of a VRE cluster and led to the implementation of interventions (staff re-education, sterile gloves, ultraviolet light disinfection) to prevent further infections [17].

Our study has a few limitations. The room environment of patients was not sampled along with the patient’s rectal swabs or blood samples. These data might have added to our understanding of VRE transmission networks across the different units of the hospital. These transmissions might have been linked to a primary colonization of patients in the same or other health centers or linked to family members or relatives. Second, isolates were collected retrospectively as they became available. Additionally, a single isolate per clinical sample was used in the analyses, potentially missing the full diversity of VRE and transmission events.

In summary, we demonstrated the emergence, spread, and persistence of drug-resistant *E*. *faecium* in immunocompromised patients over a 7-year period in a single comprehensive cancer center. Further research is needed to better understand the evolution of resistance and virulence of VRE and develop effective prevention strategies. Additionally, associations between VRE and other bacterial species are of importance as the evolution of resistance can be due to interactions between the different microbial gut flora. WGS is an essential tool that may identify or predict antibiotic resistance and virulence traits of different pathogens and hidden transmission networks in order to implement more targeted infection control and disinfection measures. As more antibiotic-resistant bacteria are emerging, it is crucial that hospitals re-evaluate their infection prevention procedures and regularly educate physicians and hospital staff members to prevent outbreaks and limit the transmission of multi-drug-resistant microorganisms.

## Figures and Tables

**Table 1:** **Sequence types of the selected 82 vancomycin-resistant enterococci strains.** Newly isolated STs compared to 2012 are highlighted in light grey. The novel ST323 is highlighted in a darker grey and the 2 STs only harboring DR-VRE strains are framed in black.

	Leukemia (n = 44)		HCT (n = 23)		ICU (n = 15)	
	DR	Non-DR	DR	Non-DR	DR	Non-DR
**Culture type**						
Rectal swab, n (%) *	1 (2)	8 (18)	5 (22)	6 (26)	0	0
VRE bacteremia, n (%)	10 (23)	25 (57)	4 (17)	8 (35)	8 (53)	7 (47)
**Sequence type**						
**Unknown, n (%) †**	1 (2)	2 (5)	0	0	0	0
Known, n (%)						
ST17	0	10 (23)	0	6 (26)	1 (7)	3 (20)
ST18	1 (2)	8 (18)	0	2 (9)	0	2 (13)
ST80	0	6 (14)	2 (9)	3 (13)	0	0
ST736	2 (5)	0	2 (9)	0	3 (20)	0
ST664	3 (7)	0	3 (13)	0	2 (13)	0
ST412	2 (5)	2 (5)	1 (4)	0	0	0
ST584	1 (2)	0	0	1 (4)	1 (7)	1 (7)
ST117	0	1 (2)	0	0	0	1 (7)
ST203	0	1 (2)	0	0	0	0
ST494	0	0	1 (4)	0	0	0
ST323	0	0	0	0	1 (7)	0
ST431	0	1 (2)	0	0	0	0
ST789	0	1 (2)	0	0	0	0
ST282	0	0	0	1 (4)	0	0
ST233	0	1 (2)	0	0	0	0
ST1390	0	0	0	1 (4)	0	0
ST1471	1 (2)	0	0	0	0	0

* Percentages are in relation to either the Leukemia, HCT, or the ICU total patient numbers. † Due to recombination events in one or more of the 7 housekeeping genes selected for VRE MLST. Abbreviations: HCT, hematopoietic cell transplant; ICU, intensive care unit; DR, daptomycin-resistant strains; STs, sequence types.

**Table 2:** Antibiotic resistance genes in the 82 vancomycin-resistant enterococcal strains.

Antibiotics	Gene	Total (n = 82)	LKM (n = 44)	HCT (n = 23)	ICU (n = 15)
**Vancomycin, n** (**%**)	*vanA*	81 (99)	44 (100)	22 (96)	15 (100)
	*vanA and vanB*	1 (1)	0	1 (4)	0
**Daptomycin, n** (**%**) *		28 (34)	11 (25)	9 (39)	8 (53)
**Linezolid, n** (**%**) †	*optrA*, *poxtA*, *cfr*, *eatAv*, 23S rDNA	11 (13)	6 (14)	4 (17)	1 (7)
**Macrolides, n** (%)	*erm(B)*	67 (82)	36 (82)	18 (78)	13 (87)
**Macrolides, lincosamides, and streptogramin B, n** (**%**)	*msr(C)*	81 (99)	44 (100)	22 (96)	15 (100)
**Teicoplanin, n** (**%**) **	*vanHAX*, *vanR/vanS*	78 (95)	43 (98)	22 (96)	13 (87)
**Tetracyclines, n** (**%**)					
**Tetracyclines only**	*tet(L)*	37 (45)	17 (39)	12 (52)	8 (53)
**Minocycline and tetracyclines**	*tet(M)*	63 (77)	35 (80)	16 (70)	12 (80)
**Aminoglycosides, n** (**%**)					
**Streptomycin**	*ant(6)-Ia*	51 (62)	31 (71)	9 (39)	11 (73)
**Kanamycin, neomycin**	*aph(3’)-III*	64 (78)	36 (82)	17 (74)	11 (73)
**Trimethoprim, n** (**%**)	*dfrG*	22 (27)	9 (21)	6 (26)	7 (47)

*Abbreviations:* LKM, leukemia; HCT, hematopoietic cell transplant; ICU, intensive care unit. * Daptomycin resistance was conferred by the presence of amino-acid substitutions in predominant LiaFSR proteins; threonine to alanine on position 120 of the LiaS protein sequence, tryptophan to cysteine on position 73 the LiaR sequence, histidine to arginine on position 215, and aspartic acid to asparagine on position 27. † Linezolid resistance was conferred by the presence of the *optrA*, *poxtA*, *and cfr* genes; mutations in the 23S rDNA genes; laboratory susceptibility testing, with a minimum inhibitory concentration range of 8–64 µg/mL. ** Teicoplanin resistance was conferred to the presence of the *vanHAX* gene operon and the VanR/VanS two-component system [18,19].

## Supplementary Materials

The following supporting information can be downloaded at: https://www.mdpi.com/article/10.3390/microorganisms12081676/s1, Figure S1: Phylogenetic tree showing the genetic relatedness according to SNPs found in the core genome of the 89 VRE isolates from 2012 (previously published [1]) and 82 VRE isolates from 2015 to 2019; Figure S2: Phylogenetic tree showing genetic relatedness according to SNPs found in the core genome of the 82 VRE isolates from 2015 to 2019 showing the source of isolation (gastrointestinal, blood) and the antibiotic resistance profiles; Figure S3: Transmission networks of DR-VRE isolates; Figure S4: Transmission networks of other VRE isolates without daptomycin resistance.
