# Supplementary material for: Persistence of Daptomycin-Resistant and Vancomycin-Resistant Enterococci in Hospitalized Patients with Underlying Malignancies: A 7-Year Follow-Up Study
Source: Microorganisms. 2024 Aug 14;12(8):1676. doi: 10.3390/microorganisms12081676 (PMC11356748; doi:10.3390/microorganisms12081676)
Supplement: Supplementary file 1 [file microorganisms-12-01676-s001.zip › microorganisms-3138614-supplementary.pdf]

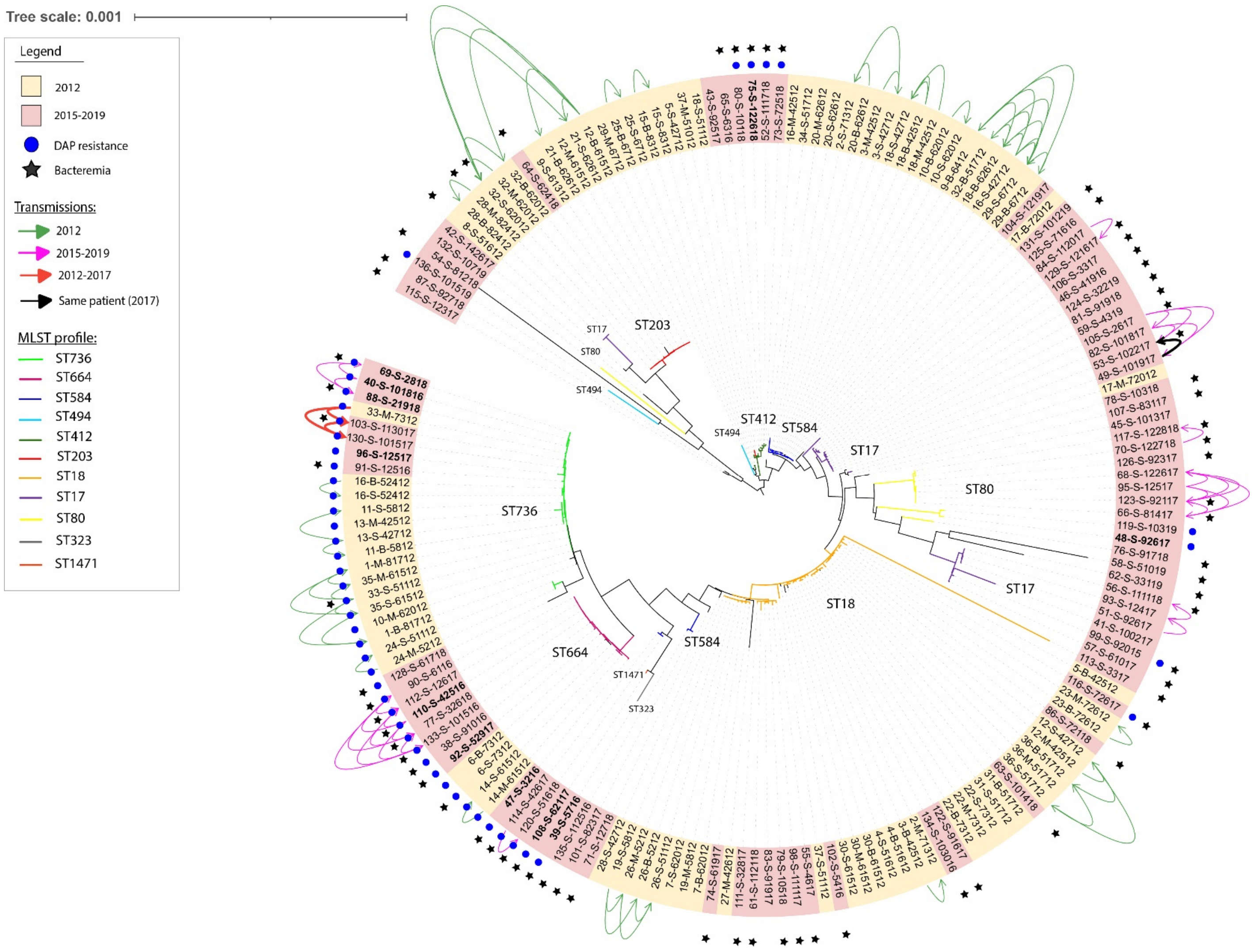

Figure S1. Phylogenetic tree showing the genetic relatedness according to SNPs found in the core genome of the 89 VRE isolates from 2012 (previously published [1]) and 82 VRE isolates from 2015 to 2019. Transmissions between patients are shown. *Abbreviations:* ST, sequence type; DAP, daptomycin; S, rectal swabs; M, main room; B, bathroom.

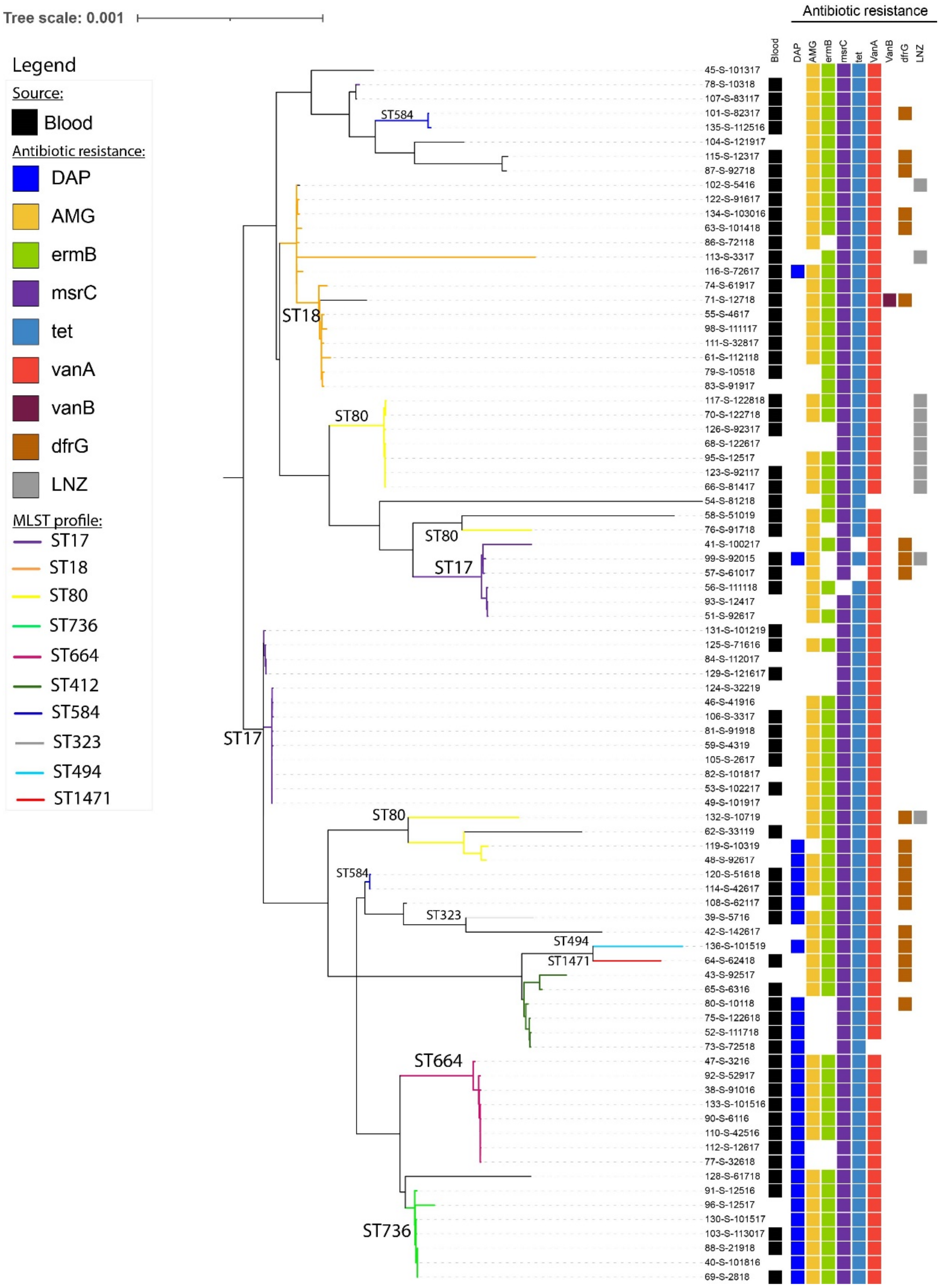

Figure S2. Phylogenetic tree showing genetic relatedness according to SNPs found in the core genome of the 82 VRE isolates from 2015 to 2019 showing the source of isolation (gastrointestinal, blood) and the antibiotic resistance profiles. *Abbreviations:* DAP, daptomycin; S, rectal swabs; AMG, aminoglycoside*; ermB,* erythromycin*; msrC,* macrolide, lincosamide, and streptogramin B*;* tet, tetracycline*;* VanA, vancomycin A*;* VanB, vancomycin B*; dfrG,* trimethoprim*;* LNZ, linezolid.

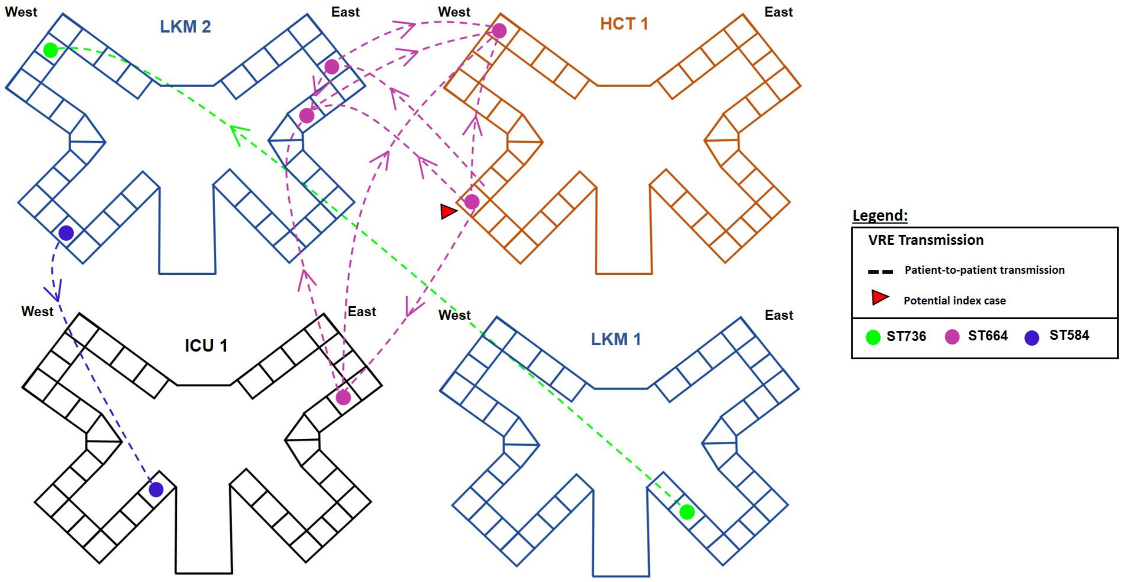

Figure S3. Transmission networks of DR-VRE isolates differing by ≤5 SNPs in their core genomes during 2015 to 2019 in the hospital setting. Potential index cases of VRE are marked with a red triangle.

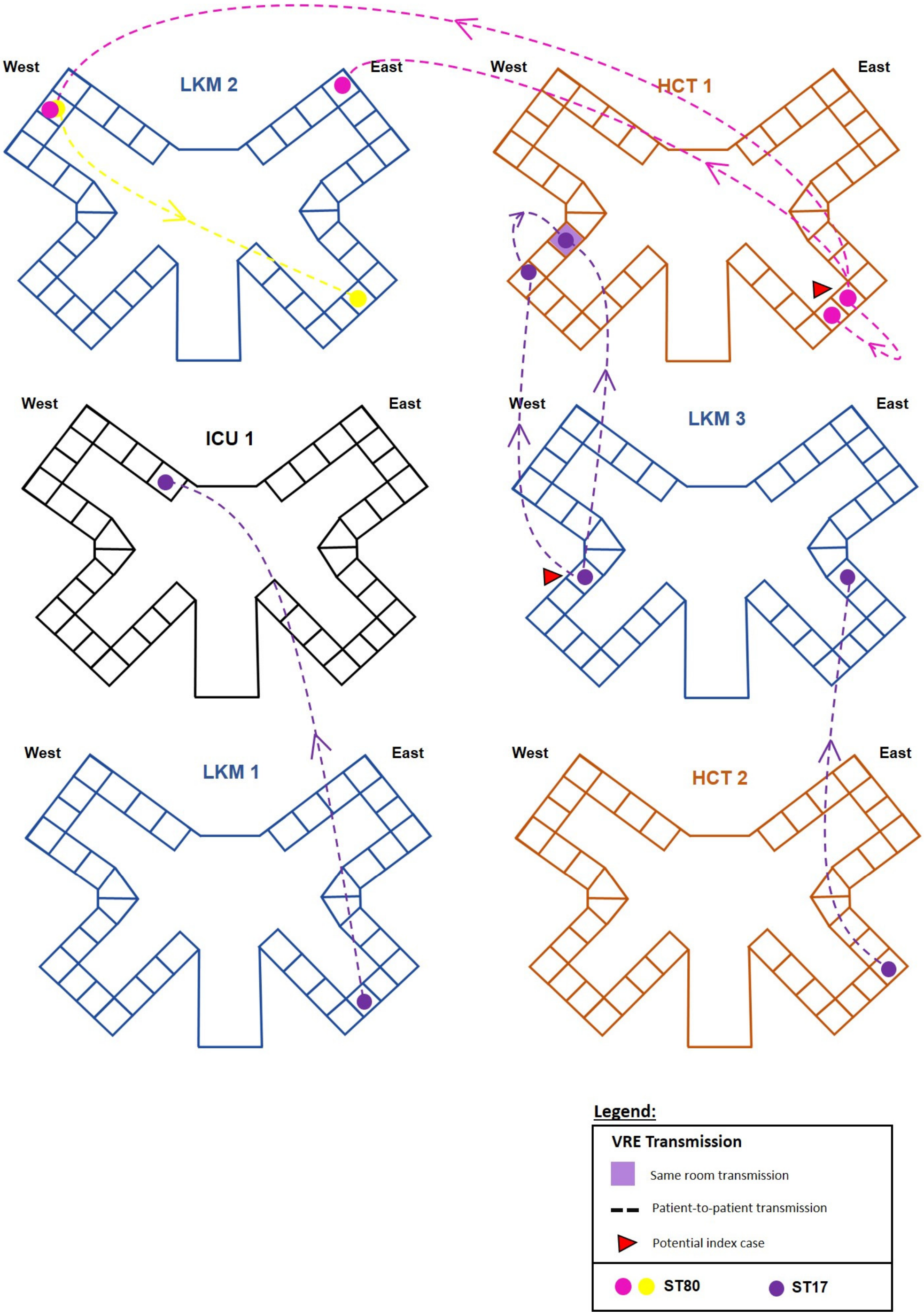

Figure S4. Transmission networks of other VRE isolates without daptomycin resistance differing by ≤5 SNPs in their core genomes during 2015 to 2019 in the hospital setting. Potential index cases of VRE are marked with a red triangle. *Note: There are 2 distinct clusters of ST80 VRE strains originating from the same room (different patients, different dates) and spread to patients on different floors, indicating possible recombination events and/or horizontal transmission.*

1. El Haddad L, Hanson BM, Arias CA, et al. Emergence and Transmission of Daptomycin and Vancomycin-Resistant *Enterococci* Between Patients and Hospital Rooms. Clin Infect Dis **2021**; 73(12): 2306-13.
